# Retrospective Comparison of Laparoscopic versus Open Radical Hysterectomy for Early-Stage Cervical Cancer in a Single Tertiary Care Institution from Lithuania between 2009 and 2019

**DOI:** 10.3390/medicina58040553

**Published:** 2022-04-17

**Authors:** Danuta Vasilevska, Dominika Vasilevska, Andrzej Semczuk, Vilius Rudaitis

**Affiliations:** 1 Department of Gynecology, Vilnius University Hospital Santaros Clinics, 08661 Vilnius, Lithuania; 2 Faculty of Medicine, Vilnius University, 03101 Vilnius, Lithuania; dominikavasilevska@gmail.com (D.V.); vilius.rudaitis@santa.lt (V.R.); 3 II^ND^ Department of Gynecology, Lublin Medical University, 20090 Lublin, Poland; andrzejsemczuk@umlub.pl

**Keywords:** early-stage cervical cancer, LACC study, radical hysterectomy, surgical gynecological oncology

## Abstract

*Background and Objectives*: A great debate within the academic arena was evoked by the LACC study, giving rise to doubt regarding the oncological outcomes of the laparoscopic approach for early-stage cervical cancer. This encouraged us to conduct a retrospective analysis of CC treatment surgical approaches applied to the patients at tertiary level Vilnius University Hospital Santaros Clinics, Vilnius, Lithuania, between 2009 and 2019. *Materials and Methods*: The retrospective study was carried out to evaluate the outcomes after 28 laparoscopic and 62 laparotomic radical hysterectomies for early cervical cancer in a single tertiary care institution performed during the period 2009–2019. For statistical analysis of patients’ parameters, SPSS v. 17.0 was applied, together with the Kaplan–Meier method with a long-rank test and the Cox proportional hazard regression model used for bi-variate analysis determining OS outcomes between MIS and open-surgery groups. *Results*: After computing data with the Cox regression model, there was no significant difference of the 36-months overall survival between laparoscopy and laparotomy groups, as opposed to the LACC study. *Conclusions*: Our tertiary institution faces a considerable challenge, and we acknowledge the limitations of the study and also feel a responsibility to follow the latest guidelines. Currently, it appears that the most substantial attention should be focused on the cessation of uterine manipulator use as well as laparoscopic technique learning curves.

## 1. Introduction

Despite the fact that the Pap smear has become widely available, that the use of human papillomavirus tests and vaccination are increasing, and that the incidence of cervical cancer (CC) has decreased, it still remains the fourth most common cause of cancer death for women, especially in developing countries [1]. CC accounts for approximately 527,600 new cases and 265,700 deaths each year globally [2]. From 1987 to 2002, mortality from cervical cancer in Lithuania increased by 2% and decreased by 2.3% annually thereafter due to screening program implementations [3]. According to the WHO International Agency for Research on Cancer statistics, in 2018, there were 431 new cases of CC (2.6% of all cancer cases) and 209 deaths (15% of deaths caused by cancer and 0.6% of all deaths) in Lithuania, making it one of the highest incidences of CC in the European Union [3].

Radical hysterectomy (RH) with pelvic lymphadenectomy remains the “golden” standard recommendation for patients with early-stage (IA2-IIA) CC [4,5,6,7,8,9]. For patients with early-stage CC, surgery remains the standard recommendation of care.

Traditionally, RH has been performed as an open surgery through a laparotomy incision. However, despite the excellent five-year overall survival (OS) rate, this approach is associated with considerable peri-operative complications and morbidity [4,8]. Starting with the introduction of laparoscopic RH in 1992, numerous retrospective studies reported that this procedure is associated with fewer early post-operative complications, earlier recovery, shorter hospital stay, decreased morbidity, reduction in blood loss, lesser bladder and wound infections, and lower transfusion rates, leading to a widespread acceptance of RH performed by minimally invasive surgery (MIS) [4,10]. Despite the benefits of MIS for CC and worldwide patient acceptance, for a long time, there was a lack of well-designed randomized clinical trials comparing laparoscopic radical hysterectomy and abdominal radical hysterectomy in terms of recurrence and overall and disease-free survival [4,8]. Despite the paucity of high-quality supporting evidence, laparoscopic RH was universally favorable until 2018, when two independent studies demonstrated a poorer outcome for women who had undergone radical surgery via a minimal access route. The Laparoscopic Approach of Carcinoma of the Cervix (LACC) trial by Ramirez et al. [5], a prospective, randomized, international, multicenter trial, has become a ground-breaking landmark event in the history of gynecological oncology, unexpectedly reporting significantly unfavorable disease-free survival and overall survival in the MIS RH group than in the open radical hysterectomy (ORH) group, showing a discrepancy between the results of this study and the majority of the data published previously [11]. The LACC trial indicates that changing from MIS RH to ORH would reduce the number of recurrences by six and the number of deaths by five out of every hundred patients. Paradoxically, the quality of MIS is equal to that of open surgery in terms of the tissue specimens. Hence, explanations should be sought as to the differences between the surgical approaches [5]. A second analysis, which included data from two national U.S. databases and was presented by Melamed et al. [8], revealed a 48% higher risk of death from any cause within 4 years after a minimally invasive radical hysterectomy and a pelvic lymphadenectomy for stages 1A2–1B1 CC [12].

The unexpected results of both studies have evoked a great debate within the academic arena [13]. Some authors notice clear bias as well as a lack of external validity in LACC trials and believe it may be a “double-edge” sword, as these results have already been published outside the medical community and will affect the practice of gynecologic oncologists, the treatment guideline of CC, as well as the patients’ attitudes [14,15,16,17]. We believe that the true impact of the LACC trial is not that it should change the standard of care across the globe, but rather that real effort should be put into understanding the results. A single-aspect approach would not benefit either patients or surgeons; hence, this issue should be analyzed through a complex and comprehensive prism.

Heavy references on the LACC trial and the significant wave encouraged us to conduct a retrospective analysis of CC treatment surgical approaches applied to the patients at the tertiary level Vilnius University Hospital Santaros Clinics, Vilnius, Lithuania, between 2009 and 2019.

## 2. Materials and Methods

We performed a retrospective observational trial comparing the survival in two patient groups with cervical cancer that were treated with abdominal RH or the MIS approach—laparoscopic RH.

Retrospective data analysis was selected to gather research information. Data regarding patients treated for diagnosis of “cervical cancer” (according to ICD—C53; C53.0; C53.1; C53.8; C53.9) in the Gynecology Department at the Vilnius University Hospital Santaros Clinics, during the period of 2009–2019, were collected from the electronic case-histories database, while survival data were obtained from the Population Register Service. Case summaries, operation protocols, as well as histopathological outcomes of 311 women were carefully reviewed.

Out of the 311 CC patients that were reviewed, the outcomes of 90 patients with stage IA2-IB2 CC (stage was based on FIGO 2009), who were treated with the primary radical surgical treatment, were analyzed. Although ESGO recommendations indicate that stage IIA is also operable, we only included patients with FIGO stage I CC in order to prevent potential distortions of the results. In fact, out of 311 patients, 221 were excluded from further analysis, as the majority of patients were not eligible for radical hysterectomy due to advanced disease, older age, second primary tumor, high body mass index, surgery was not type C radical hysterectomy (due to undiagnosed cervical cancer at the beginning of treatment), and, in some cases, a histopathological examination answer was not found in the electronic case-histories database or the operation was performed recently, thus making it impossible to calculate the 36-months OS (only patients who had undergone operation until 2019 were included).

The following variables were included in the statistical analysis: patients’ age, surgical approach, operation type, tumor histologic type, level of differentiation, LVSI, parametrial involvement, positive margins, extent of lymphadenectomy, number of positive lymph nodes, operation time, blood loss, postoperative complications, stage of the disease proven by histopathological examination, and the 36-months OS. In order to treat early-stage (IA1-IB2) CC, primary surgery for 90 patients was performed, carrying out a radical C1 hysterectomy with regional lymphadenectomy. For 62 patients (68.9%), RH was performed by the open-surgery method, while for 28 women (31.1%), MIS RH was chosen. The decision on surgical approach mostly depended on the patient‘s preference and surgeon’s experience at the certain point of time. This fact is supported by further presented patients‘ characteristic analysis in both groups that did not show any statistically significant differences. For all patients in the MIS group, the vaginal creation of a tumor covering vaginal cuff and the strict avoidance of use of any uterine manipulator was applied.

Statistical analysis of clinicopathologic parameters between the patients who underwent MIS RH and those who underwent ORH was performed applying the SPSS v. 17.0 (IBM SPSS Statistics, IBM Corporation, Chicago, IL, USA) and R Version 3.5.3 (R Core Team (2019), R Foundation for Statistical Computing, Vienna, Austria) statistical packages and Microsoft Office Excel 2016. First, the normality of the gathered continuous variables was tested using the Kolmogorov–Smirnov test (for subgroups with more than 50 subjects) and Shapiro–Wilk test (for subgroups with less than 50 subjects). If the *p* value was <0.05, a non-parametric Mann–Whitney U test was applied; however, if *p* > 0.05, the parametric Student’s t-test was performed. Frequency distribution between the categorical variables was compared using Pearson’s chi-squared test and Fisher’s exact test (if expected values were less than 5). For normally distributed quantitative variables, the mean ± standard deviation was calculated; otherwise, the median, minimum, and maximum values were calculated. The strength of the association between events in MIS and open-surgery groups was quantified with the odds ratio (OR) and 95% confidence intervals (95% CI). The Kaplan–Meier method with a long-rank test and the Cox proportional hazard regression model, together with the hazard ratio and 95% confidence intervals, were used for bi-variate analysis determining the OS outcomes between MIS and open-surgery groups and data visualization in survival curves. Statistical significance was defined when *p* was less than 0.05.

## 3. Results

### 3.1. Subjects’ Disease and Tumor Characteristics

Detailed information on subjects’ disease and tumor characteristics are presented in Table 1. Frequency of conization prior to surgery was similar in both groups. Squamous cell carcinoma was the most frequent type of CC detected during the histopathological examination of biopsy or conization in both groups. The clinical stage of CC (FIGO stage) was determined according to bimanual pelvic examination, histopathological verification from biopsy/conization material, and radiological imaging, such as CT, MRI, or PET CT. Before surgery, the most considerable number of patients were diagnosed with stage IB1. If no residual tumor was found, it was interpreted that all the tumor was removed during conization. Poorly differentiated (G3) CC was the most prevalent among CC grades in both groups. In both groups, the tumor size was above 2 cm in diameter, although it was truly difficult to evaluate the tumor size or volume due to procedures (conization and biopsy) performed previously; hence, the precise tumor size lacks in about one-third of the cases.

Although deep stromal invasion and positive LVSI were much more frequent in the MIS group than in the open-surgery group, it showed no statistical significance. There were no positive margins in either group. In all patients, pelvic lymphonodectomy was performed, while part of the para-aortic lymph nodes was excised for 12.1% of women in the open-surgery group (if pelvic lymph nodes were positive from frozen specimens during the surgery). The medians of the removed lymph nodes in the MIS group were 11 and 19 in the open-surgery group—these are the only results to show a significant difference between groups. Nevertheless, only one patient in the MIS group showed positive lymph nodes in the final pathological review, whereas almost 18% of women in the open-surgery group were diagnosed with positive lymph nodes. In terms of the administration of adjuvant radiotherapy, it was considerably more frequent in the open-surgery group; however, it was not statistically significant.

### 3.2. Characteristics of MIS and Open Surgery 

Comparing the duration of surgery, the mean time for RH with the laparoscopic approach was 218 min ± 39 min, while for the open surgery, it was slightly shorter (205 min ± 42 min). Concerning short-term postoperative complications (i.e., fever and anemia), there was a lower risk after undergoing laparoscopic surgery. A statistically significant difference was observed while analyzing the length of hospital stay—it was shortened 1.5 times after MIS RH compared to recovery after open-surgery operation (shown in Table 2).

### 3.3. Patients’ OS Analysis

**Figure 1:**
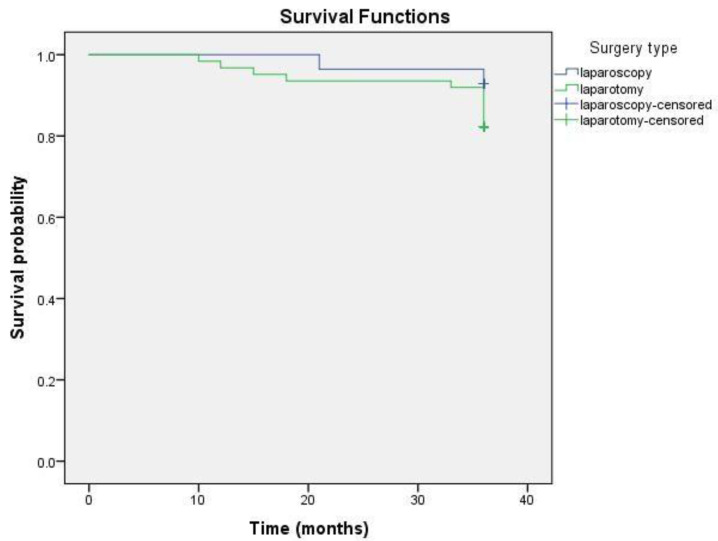
Kaplan–Meier 3-year OS curve of CC patients who underwent the laparoscopic or laparotomic surgical approach for RH.

**Figure 2:**
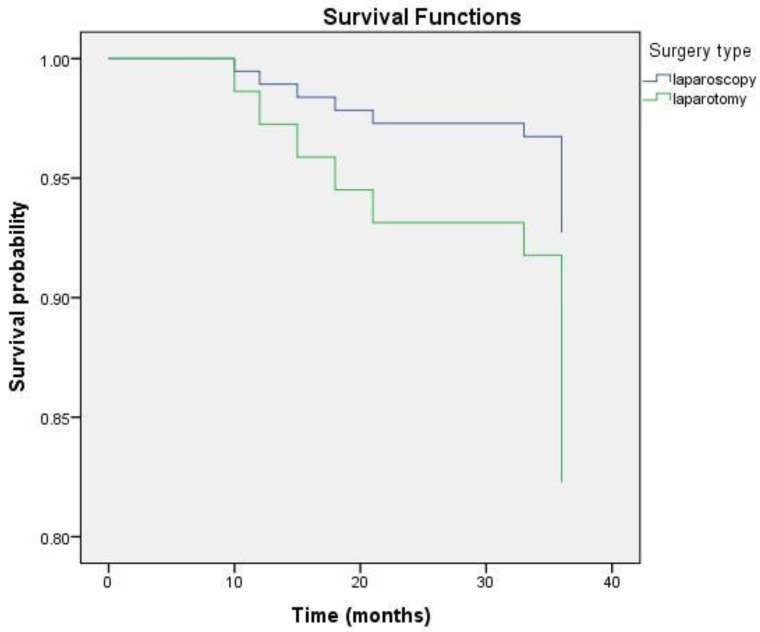
Cox (proportional hazards) regression curves for 3-year OS between laparoscopic and laparotomic surgical approach for RH.

The 36-months OS was compared between two groups. The Kaplan–Meier test revealed that the OS for the whole group was 34.81 ± 0.51 months (95% CI = 33.81–35.82). On average, the survival duration in the laparoscopic group was 35.46 ± 0.53 months, while in the open-surgery group, it was 34.52 ± 0.7 months (Figure 1). Statistical analysis applying the long-rank test did not show a significant difference (*p* = 0.426). During the three-year period after surgery, one woman (3.57%) in the MIS group and five women (8.06%) in the open-surgery group died. After computing these data with the Cox regression model, the results showed that laparoscopic treatment may have a lower risk of mortality and longer survival, although *p* = 0.44, HR = 0.429, and 95% CI = 0.05–3.67, there was no statistical significance (Figure 2). Thus, there was no significant difference in the three-year OS between the laparoscopy and laparotomy groups.

## 4. Discussion

The results of the LACC trial, announced at the Society of Gynecologic Oncology (SGO) 2018 annual meeting, have attracted significant attention [18]. Due to the enormous difference in survival in favor of open surgery, this trial was stopped prematurely. In comparison, MIS has been shown to have better surgical outcomes with equivalent survival rates in patients affected by endometrial, colorectal, and gastric cancers in previously randomized trials. 

In the retrospective analysis using the Surveillance, Epidemiology, and End Results (SEER) data of the National Cancer Institute (NCI) in the US, the introduction of MIS correlated with a decrease in survival rate due to CC, thus further corroborating the results of the LACC trial [8]. According to our results, there is no difference in OS between the MIS and ORH groups; in addition, MIS may benefit patients while experiencing less short-term postoperative complications and a quicker recovery time.

The effectiveness and adequateness of MIS RH in terms of surgical and oncological outcomes have been described in numerous reports [18,19,20,21,22]. Not all data presented in the post-LACC era confirmed an unfavorable oncologic outcome with MIS. For instance, a recent cohort study from Denmark, another study from the Memorial Sloan-Kettering Cancer Center, and a retrospective analysis of the database of nine French university hospitals demonstrated similar survival outcomes between MIS and open surgery [23,24]. In contrast, a recent large multicenter cohort study (including centers from Latin America and Europe), evaluating the long-term outcomes of laparoscopy for early-stage CC, supported the LACC trial results [25]. In another study, similarly to the LACC trial, a tendency towards earlier recurrences in the MIS group was observed. However, with an extended follow up, the disease-free survival (DFS) and OS curves of laparoscopic and open procedures overlapped, highlighting the feasibility, safety, and improvement in the post-operative functional outcomes of the laparoscopic nerve-sparing radical hysterectomy [26]. A recently published meta-analysis of 50 studies supports the idea of poorer DFS in the MIS group [27].

NCCN guidelines updated in 2019 recommended only open surgery for CC treatment, putting us in doubt and challenging the management of CC in our tertiary level hospital. MIS is suggested to be applied in the case of tumors of less than 2 cm, as the laparoscopic approach showed 5-year OS and DFS rates similar to those of an abdominal hysterectomy, as opposed to patients with tumor sizes of ≥2 cm and <4 cm, where MIS showed lower DFS [2,8,23,28,29,30]. However, there are two studies with different results, emphasizing the likelihood of disease recurrence in a patient group with CC < 2 cm [29]. From 2019, our tertiary level hospital has completely turned away from MIS RH for CC treatment.

A survey was conducted among ESGO members, revealing that 57% of respondents have changed their approach to open surgery, while 50% perceive MIS to be appropriate only for small tumors [13]. It is claimed that the primary disadvantages of the laparoscopic technique are at a higher rate of disease-positive surgical margins, resulting in the need for adjuvant therapy and the slow learning curve required for a surgeon to gain precise skills. In contrast, results obtained in this retrospective analysis do not prove the former assumption [2].

Results obtained in our study as well as by others suggest that women who underwent MIS are similar to those in the open-surgery group in terms of histopathological variables (differentiation grade, histological subtype, parametrial invasion, positive margins, lymph nodes involvements, and LVSI) [8,18,25,31]. However, these data could be misinterpreted as conization prior to the surgery and may falsify the existing results. The LACC trial showed a statistically significant hazard ratio for lymph node involvement and LVSI, and adenocarcinoma was regarded as an unfavorable prognosticator [2,32,33]. Considering the results of Melamed et al. [8], it is notable that their methods, sample selection criteria, and particular results are similar to ours; however, there is disparity in survival (MIS group shows a poorer outcome). In the LACC study, the quality of the MIS was almost the same as that of open surgery in terms of tissue specimens. This means it is more probable that there is an inequality between the surgical procedures themselves, not in the subjects, a tumor or disease characteristic. First, a possible speculation explaining these inequalities could be the use of uterine manipulators, which are believed to have a squeezing effect disseminating the tumor cells during MIS RH [34,35]. In contrast to the study of Melamed et al. [8], a uterine manipulator was never used in radical hysterectomy in our hospital (we completely closed the cervix with a vaginal cuff prior to laparoscopy). It could be speculated that this fact was the main reason for the operation time difference between the two groups, as it takes approximately 20–30 min to develop a vaginal cuff and to completely close the cervix. Several authors proved that uterine manipulators are safe for endometrial cancer (EC) laparoscopic surgery; however, their application for CC surgery remains controversial [12,16,36,37,38,39,40,41]. Second, a common belief exists that surgeons still have not mastered MIS skills properly, thus making too many additional manipulations during the surgery and disseminating the tumor or even not resecting the margins radically. Although it may be considered as a drawback, all operations analyzed in our study were performed by one surgeon, which allowed us to compare the outcomes without a larger probability of technique factor impacts. It is anticipated that the mastery of laparoscopic RH requires experience of at least 25 to 50 cases, which means that the optimal surgical outcomes of MIS for CC, which was broadly adopted in 2006, are just now being introduced [12,42,43]. Moreover, the data of two video cases were not sufficient for a surgeon’s recruitment to the LACC trial, and there was a lack of objective assessment, raising the question as to whether all participating surgeons had sufficient operational experience [15,31,44]. There is a high probability that the radicality of surgery was not fully achieved through MIS in the LACC trial [16]. Which specific improvements during the learning curve account for the increased survival is unclear, but the reduction in complications that could delay adjuvant treatment may be the answer [41]. In sum, there is evidence that a laparoscopic radical hysterectomy is safe without using a uterine manipulator for stage IB CC, in the hands of well-trained surgeons [39,45].

There is no actual report of manipulator application in the LACC trial; however, this study tends to believe that the effect of the insufflation gas (CO_2_) on tumor-cell growth is more considerable. Circulating pneumo-peritoneum gas disturbs the mesothelial layer and may provoke cancer cell implantation and initiate inflammatory processes [10,12,46]. It is also believed that a total laparoscopic intracorporeal colpotomy under CO_2_ possesses a risk of a positive vaginal cuff margin [47]. Moreover, fragmentation of metastatic nodes during MIS may lead to peritoneal seeding [11,48,49]. On the other hand, squamous-cell carcinoma is believed to have a lower rate of ovarian metastases and lymph node metastases with less peritoneal implantation and hematogenous dissemination. In consequence, this may reduce the effect of CO_2_ pneumo-peritoneum to some extent [28]. Another peculiarity of MIS is the steep Trendelenburg position, which may be linked with fluid or tumor cell accumulation in the upper abdomen [2,16]. MIS is thought to have inherent limits in the extent of the resection, as the instruments function only at a particular angle (i.e., narrower resection of uterosacral ligaments or parametria) [44]. In the LACC study, the vault was shown to be the most common site of recurrence for open surgery, whereas it was the pelvis for MIS. However, in gastric, colon, cystic, prostate, and epithelial ovarian cancer, similar oncologic outcomes were obtained in the open-surgery and MIS groups [13,50]. These cancers did not involve direct tumor handling; thus, the uterine manipulator, not the pneumo-peritoneum gas, may have a more considerable role in disseminating the tumor cells. However, some authors argue that a well-designed randomized trial could reveal similar results for these cancers as for CC [51].

Conization is estimated to be a favorable prognostic factor for OS. In our study, there was no distinction in outcomes between patients with and without conization performed prior to the surgery. Some authors argue that histopathological examination of the conization sample may help to accurately improve the treatment plan as well as to reduce the potential spillage of tumor cells during the surgery due to the reduced volume of the tumor [2,52]. Others argue that it is a “double-edge” sword, as after the surgery, when conization was performed before, it is difficult to obtain a whole image of the disease and tumor from the histopathological examination [15]. 

There is a growing amount of evidence that MRI outweighs CT in terms of the evaluation of patients with CC before surgery. We recognized that MRI was performed only in a few patients in this study; hence, our tertiary care hospital was not able to participate in the European Cohort Observational Study (SUCCOR). We are encouraged to reconsider the standards of patients’ radiological assessment and implement a mandatory MRI scan in the near future.

The results of the SUCCOR study comparing patients with stage IB1 CC showed that MIS RH possesses a significantly higher risk of relapse and death. However, the survival was similar in the subgroups of patients with tumors <2 cm and in patients who underwent MIS RH without a manipulator [53].

The addition of adjuvant therapy to surgery is considered to be a predictor for more postoperative complications, poorer life quality, and an increased morbidity rate [54,55,56]. Thus, keeping in mind all risk features and considering the need for adjuvant therapy after surgery, in terms of statistical significance, both investigated groups are coequal. One of our patients in the MIS group received postoperative chemotherapy due to the fact that, before and during surgery, there was an unrecognized small endocervical adenocarcinoma metastatic lesion in one of her ovaries. She developed a progression of disease in the liver and lungs and died in the 19th postoperative month. After this event, we included PET-CT into our preoperative radiological imaging protocol in cases of high-risk cervical cancer.

It is worth it to separately note Korean data [4], which show even better results in the MIS group in terms of a lower frequency of complications, shorter hospital stays, less adjuvant therapy, reduced medical costs, and the most noteworthy, better overall survival compared to abdominal operations. MIS is more widespread in Korea than in other countries; for instance, in 2014, as high as 51.8% of RH in Korea were performed by MIS compared to only 15% in the USA during the period 2006–2010 [2,4,57]. This elevated trend is attributed to patients’ demands and preferences as well as better cosmetic effects. It accelerates competition among surgeons; hence, they are constantly improving their skills. However, it is criticized that in these cohorts, laparoscopic RH was performed only for small-volume tumors, leading to better survival outcomes and a lower need for adjuvant therapies. In contrast, some studies conducted in Korea support the LACC trial and prove that MIS RH is inferior to ORH in terms of survival [2,11].

Survival in the UK cohort, similarly to our results, showed no difference in mortality rates in terms of surgical approach [31]. Nonetheless, this study and some other studies found an association between tumors of more than 2 cm and positive LVSI and patients’ mortality [2,8,31,58]. Where there was no contrast in OS between the two surgical methods in patients with a tumor size of less than 2 cm, there was better survival in the ORH group in patients with tumors of 2–4 cm in size. On the contrary, our study together with the LACC trial and Cusimano et al. [45] showed a similar mortality hazard for tumors of all sizes. Thus, the critical role of tumor size should be reevaluated when determining surgical modality, and the oncological importance of tumors of less than 2 cm should be rethought when designing future studies.

Our study, as well as the LACC trial, may have inadequately analyzed the outcomes between subgroups; for instance, it could be determined in more detail which tumor size and stage, histologic type, and age groups experienced more considerable recurrence and mortality rates. We admit that our limitations may be the retrospective approach to data analysis, the single tertiary hospital, and small patient groups. However, it could be claimed that, in some cases, retrospective analyses gave a more realistic image than randomized trials, which represent only a fraction of all eligible patients, worsening the external validity of the study. We recognize that a three-year patient follow up is a limited period of time to draw objective conclusions; however, a longer follow up would mean significantly reduced patient groups, though we are willing to conduct a similar study in a few years with more eligible cases. On the other hand, our personal experience, as well as scientific reports, highlight that the majority of recurrences occur within two years of treatment [59].

Recently, two large randomized clinical trials for early-stage CCs have been launched (the Chinese trial and the International Robot-Assisted Approach to Cervical Cancer (RACC) trial). The results of these trials are not expected until 2025; therefore, it is believed that the LACC trial data may dominate the academic dispute and guidelines recommendation until that time [24].

## 5. Conclusions

Facing significant academic dispute concerning the surgical treatment of early-stage CC, it is important to note that study populations might be heterogeneous between subgroups in different studies, selection bias may occur, and different criteria and models could be applied by individual investigators, causing misinterpretations when comparing distinct reports. Our tertiary institution results face a considerable challenge, as our calculated survival rates of early CC do not differ between open surgery and MIS groups, although we acknowledge some limitations highlighted earlier and also feel a responsibility to follow the latest guidelines. Following them, at our institution, we choose the open approach for hysterectomy in patients with early-stage CC. However, keeping in mind the results of this retrospective study, we are encouraged to find patients who may benefit from the MIS approach. The role of MIS in early-stage CC diagnostic/prognostic approaches will certainly have to be re-evaluated, but the key point is to neither overreact to the LACC results nor ignore them. Precautions should be implemented immediately to avoid tumor cell disseminations during MIS, and patients ought to be scrupulously selected for the MIS approach together with highly qualified surgeons, whose skills have been objectively assessed. This highlights the need for more randomized trials with large comparative samples and also encourages us to continue further research with more considerable study samples and a longer follow-up period.

## Figures and Tables

**Table 1:** Patient disease, tumor, and treatment characteristics considering MIS and open-surgery groups.

Characteristic	Group by Operation Type		*p* Value
	MIS (*n* = 28)	Open-Surgery (*n* = 62)	
Age (in years)			
- Mean and standard deviation - Median	40.07 ± 8.08	43.98 ± 13.07	0.087
	39	41	0.366
Conization prior to surgery	13 (46.4%)	35 (56.4%)	0.378
Histopathological type of CC			0.113
- Squamous cell carcinoma - Adenocarcinoma - Squamous adenocarcinoma - Other	20 (71.4%)	47 (75.8%)	
	7 (25.0%)	7 (11.3%)	
	1 (3.6%)	3 (4.8%)	
	0 (0%)	5 (8.1%)	
Radiological examination			0.019
- CT - MRI - PET CT	28 (100%)	62 (100%)	
	1 (3.6%)	2 (3.2%)	
	0 (0%)	3 (4.8%)	
FIGO stage			0.599
- IA1 (LVI) - IA2 (LVI) - IB1 - IB2	3 (10.7%)	1 (1.6%)	
	0 (0%)	14 (22.54%)	
	25 (89.3%)	43 (69.4%)	
	0 (0%)	4 (6.5%)	
Hysterectomy type			0.264
- C	28 (100%)	62 (100%)	
Tumor differentiation grade			0.065
- G1 - G2 - G3	3 (10.7%)	0 (0%)	
	5 (17.9%)	18 (29.0%)	
	20 (71.4%)	44 (71%)	
Tumor size			0.086
- <2 cm - ≥2 cm - Not recorded	4 (14.3%)	16 (25.8%)	
	12 (42.9%)	25 (40.3%)	
	12 (42.9%)	21 (33.9%)	
Stromal invasion			0.193
- Superficial - Middle - Deep	0 (0%)	8 (12.9%)	
	6 (21.4%)	14 (22.6%)	
	22 (78.6%)	40 (64.5%)	
Lymphovascular space invasion			0.453
- Positive - Negative	9 (32.1%)	14 (22.6%)	
	19 (67.9%)	48 (77.4%)	
Positive margins	0 (0%)	0 (0%)	0.054
Pelvis lymph nodes resected	28 (100%)	62 (100%)	0.169
Para-aortic lymph nodes resected	0 (0%)	8 (12.1%)	0.054
Median of resected lymph nodes (range)	11 (8–25)	19 (2–54)	<0.001
Subjects with positive lymph nodes	1 (3.6%)	11 (17.7%)	0.095
Adjuvant therapy	7 (25%)	27 (43.5%)	0.093
Type of adjuvant therapy			0.19
- Radiotherapy - Chemotherapy - Chemoradiotherapy	5 patients	18 patients	
	1 patient	0 patients	
	1 patient	9 patients	

**Table 2:** Comparison of laparoscopy and laparotomy features for RH.

Characteristic	Group by Surgery Type		*p* Value
	MIS (*n* = 28)	Open Surgery (*n* = 62)	
Operation length *	218 min ± 39 min	205 min ± 42 min	0.195
Length of hospitalization stay **	6 days (3, 16)	10 days (6, 36)	<0.001
Short-term postoperative complication	5 (17.9%)	16 (25.8%)	0.205

* Mean ± standard deviation; ** median (min, max).

## Data Availability

The data supporting reported results of this study are available upon reasonable request to the corresponding author.

## Abbreviations

CC	cervical cancer
CI	confidence intervals
CT	computed tomography
DFS	disease-free survival
EC	endometrial cancer
ESGO	European Society of Gynaecological Oncology
FIGO	The International Federation of Gynecology and Obstetrics
ICD	International Classification of Diseases
LACC	Laparoscopic Approach of Carcinoma of the Cervix
LVSI	lymphovascular space invasion
MIS	minimally invasive surgery
MRI	magnetic resonance imaging
NCI	National Cancer Institute
OR	odds ratio
ORH	open radical hysterectomy
OS	overall survival
PET-CT	positron emission tomography-computed tomography
RACC	Robot-Assisted Approach to Cervical Cancer
RH	radical hysterectomy
SEER	Surveillance, Epidemiology, and End Results
SGO	Society of Gynecologic Oncology
SUCCOR	Surgery in Cervical Cancer Comparing Different Surgical Aproaches in Stage IB1 Cervical Cancer
